# Antimicrobial Photodynamic Therapy for Methicillin-Resistant *Staphylococcus aureus* Infection

**DOI:** 10.1155/2013/159157

**Published:** 2013-02-28

**Authors:** Xiu-jun Fu, Yong Fang, Min Yao

**Affiliations:** ^1^Department of Burns and Plastic Surgery, No. 3 People's Hospital, and Institute of Traumatic Medicine, School of Medicine, Shanghai Jiao Tong University, 280 Mohe Road, Shanghai 201900, China; ^2^Wellman Center for Photomedicine, Department of Dermatology, Massachusetts General Hospital, Harvard Medical School, Boston, MA 02114, USA

## Abstract

Nowadays methicillin-resistant *Staphylococcus aureus* (MRSA) is one of the most common multidrug resistant bacteria both in hospitals and in the community. In the last two decades, there has been growing concern about the increasing resistance to MRSA of the most potent antibiotic glycopeptides. MRSA infection poses a serious problem for physicians and their patients. Photosensitizer-mediated antimicrobial photodynamic therapy (PDT) appears to be a promising and innovative approach for treating multidrug resistant infection. In spite of encouraging reports of the use of antimicrobial PDT to inactivate MRSA in large in vitro studies, there are only few in vivo studies. Therefore, applying PDT in the clinic for MRSA infection is still a long way off.

## 1. Introduction

Methicillin-resistant *Staphylococcus aureus* (MRSA) was first reported in 1961 [1], and since then MRSA has undergone rapid evolutionary changes and epidemiologic expansion. The problem of MRSA infection has rapidly grown in these years. Currently, MRSA results in more than one-half of the nosocomial infections with *S. aureus* strains in most countries [2]. MRSA accounts for approximately 60% of clinical *S. aureus* strains isolated from intensive care units in the United States [3]. Most people acquire MRSA in a hospital setting (HA-MRSA). These strains establish an ecological niche in the hospital environment and are easily transmitted between patients and from doctor to patient [4]. In recent years, community-acquired MRSA (CA-MRSA) strains have emerged, where they are rapidly becoming the dominant pathogens in the community [5]. 

MRSA has altered penicillin-binding proteins (PBPs) with reduced affinity to penicillin and other available *β*-lactam antibiotics [6]. For a long time, glycopeptide antibiotics, especially Vancomycin, were extensively used in clinical practice. In the last two decades, there has also been growing concern about the increasing glycopeptide minimum inhibitory concentrations (MICs) for MRSA [7, 8]. Therefore, MRSA poses a serious problem for clinicians and patients. Due to the limited therapeutic options, infections caused by these resistant strains are usually difficult to treat. The problem of a relatively rapid acquisition of antibiotic resistance of MRSA is complicated by the relatively long-time period needed for the development of antibiotics with new mechanisms of action. As it can be anticipated that the development of resistance will continue in the coming years, it is just a question of time until the bacterium develops resistance towards newly developed antibiotics. Therefore, the necessity exists for an immediate and continual search for alternative methods against MRSA towards which no resistance can develop. One of the most promising and innovative approaches in this respect is antimicrobial photodynamic therapy (PDT) [9–11]. This therapeutic approach involves the administration of a photosensitizer, usually a porphyrin-based compound, which, upon photoactivation with visible light of appropriate wavelength, generates reactive oxygen species (ROS), such as singlet oxygen and free radicals, which are cytotoxic to bacterial cells.

This paper summarizes the mechanism of antimicrobial PDT and the progress of preclinical studies of antimicrobial PDT towards MRSA and identifies the potential applications to MRSA infection that may become valuable in the clinic.

## 2. Mechanisms of Antimicrobial PDT

**Figure 1:**
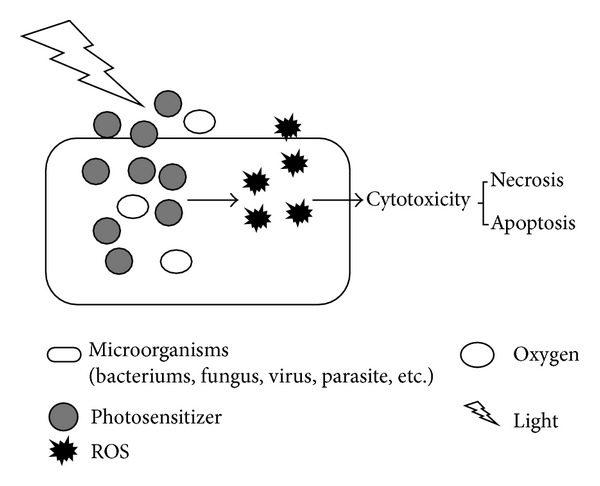
The mechanism of antibacterial PDT. Photosensitizers can be preferentially uptaken by bacteria, accumulating inside the bacteria and in the cytoplasm membranes, or in the vicinity. Upon absorption of a photon by the ground-state photosensitizer after light illumination, the reactive oxygen species (ROS) will be generated from two alternative pathways: type I mechanism and type II mechanism. The generated ROS then react rapidly with their environment depending on the localization of the excited photosensitizer: bacteria cell wall, lipid membranes, proteins and enzymes, and nucleic acids. The reaction of these important cellular components may result in necrosis or apoptosis of the bacteria at last.

Although the exact mechanism of PDT is not known in detail, there are two possible molecular mechanisms that are believed to play central roles in antimicrobial PDT. Both mechanisms cannot preclude the prerequisites for PDT: the sufficient presence of molecular oxygen, photosensitizer, and light of the appropriate wavelength. In the type I mechanism, free radicals are formed that react with lipids and proteins leading to a chain reaction that produces more oxidation products [12]. In the type II mechanism, energy from the triplet state of the photosensitizer, formed by light excitation, is transferred to the molecular oxygen, resulting in the generation of highly reactive singlet oxygen. The singlet oxygen can directly react with cellular molecules in its immediate vicinity and also creates further oxygen radicals [13]. It is generally accepted that the production of singlet oxygen plays the key role in PDT for infection and other diseases [11]. The ROS from both mechanisms react inside the bacterial cell or in vicinity and induce necrosis or apoptosis of bacteria (Figure 1).

ROS from photosensitizer-mediated antibacterial therapy can cause bacterial lethal injury by means of damage to DNA and the cytoplasmic membrane. Treatment of bacteria with various photosensitizers and light leads to both single- and double-stranded DNA break-in and the disappearance of the plasmid supercoiled fraction, which has been detected in both Gram-positive and Gram-negative species after PDT [14, 15]. Some photosensitizers that more easily intercalate into double-stranded DNA can cause more damage [16]. Evidence also shows that guanine residues of DNA are the most susceptible to oxidation by ROS [16]. However, DNA damage might not be the prime reason for bacterial cell death, because the damage may be able to be repaired by various DNA repairing systems [17]. Due to the usually lipophilic nature of many photosensitizers, they tend to locate primarily in membranes consisting of lipid double layers. Therefore, another critical damage site by ROS during PDT is the cytoplasmic membrane, which allows leakage of cellular contents or inactivation of membrane transport systems and enzymes. The alterations of cytoplasmic membrane proteins, disturbance of cell-wall synthesis and the appearance of a multilamellar structure near the septum of dividing cells, and loss of potassium ions from the cells have been reported [18–20].

The photosensitizer is the key component in the photosensitization process because it absorbs light and initiates formation of toxic species. Photosensitizers are mainly from the following classes: porphyrins, chlorines, phthalocyanine, Rose Bengal, phenothiazines, and acridines. The structures of porphyrins, chlorines, and phthalocyanine are based on the tetrapyrrole nucleus, whereas the others have different molecular frameworks [21]. These photosensitizers induce varying photodynamic activities towards Gram-positive and Gram-negative bacteria [21]. Due to structural differences of the outer bacterial cell wall of Gram-positive and Gram-negative bacteria, differences naturally exist with respect to the efficacy of the various photosensitizers. The 40–80 nm thick outer cell wall and up to 100 peptidoglycan layers of Gram-positive bacteria do not represent an effective permeability barrier. In contrast, the outer membrane of Gram-negative bacteria with a bilamellar membrane covering the only 3 nm thick peptidoglycan layer is able to impede photosensitizer diffusion considerably, especially the negatively charged or neutral photosensitizers. Various strategies have been developed to cross this barrier, such as pretreatment with EDTA or polymyxin B, which make the outer wall of bacteria more permeable and allow photosensitizer to penetrate and accumulate on the cytoplasmic membrane [22, 23]. In contrast to the low penetration of negatively charged and neutral photosensitizers, the positively charged photosensitizers are photodynamically active even without the addition of a penetration booster [24–26]. Not only resting or vegetative cells but also Bacillus spores have been shown to be inactivated using photodynamic administration [27]. As a result of the high reactivity of singlet oxygen with proteins, its lifespan in a cellular environment is very short, which results in a very short diffusion distance. Therefore, the effectiveness of a photosensitizer depends not only on the amount taken up, but also on the location of the photosensitizer at the time point of irradiation [28]. 

## 3. Inactivating MRSA by PDT

With various photosensitizers and the appropriate wavelength light, MRSA has been observed to be dramatically inactivated in a serial of in vitro studies. Wilson and Pratten [29] found that cultured MRSA was inactivated significantly by aluminum disulphonated phthalocyanine and light even in the presence of horse serum. Eight isolates of MRSA from patients were demonstrated to be completely eradicated following 15 min exposure to a 632.8 nm HeNe laser in the presence of 50 *μ*g/mL photosensitizer toluidine blue O (TBO) under in vitro conditions [30]. No significant effect was observed on the MRSA isolates exposed to the laser alone. In another study [31], light-activated antimicrobial agent aluminium disulphonated phthalocyanine (AlPcS 2) was used to determine whether 16 epidemic MRSA strains could be inactivated by antimicrobial PDT. The results indicated that all 16 strains were susceptible to inactivating by PDT. The bactericidal effect was dependent on the AlPcS 2 concentration and the light dose, and inactivation was not affected by the growth phase of the organism. Scavengers of singlet oxygen and free radicals protected the bacteria from inactivation [31].

For better simulating in vivo condition, an artificial skin construct was applied to test whether methylene blue (MB) mediating PDT could inactivate MRSA growing on it [32]. The artificial skin was composed of human-derived epidermal keratinocytes and dermal fibroblasts cultured at an air/media interface to form a stratified model of full thickness epithelialized human skin. PDT combined with MB treatment produced a significant reduction (5.1 logs) from control immediately after treatment and the effect was sustained over multiple days, while application of MB alone resulted in small reduction in MRSA viability from nontreated control [32]. 

In another study, penetration and antibacterial efficacy of a cationic porphyrin photosensitizer XF73 against MRSA was examined on an ex vivo porcine skin model [33]. The researchers performed both preincubation of bacteria in solution with XF73 followed by subsequent application on the ex vivo porcine skin and application of bacteria on the skin followed by an incubation with XF73. The localization of XF73 was restricted to the stratum corneum. Preincubation of *S. aureus* demonstrated a high photoinactivation efficacy (>3 logs reduction) after irradiation, while illumination after XF73 was delivered to the bacteria on the skin resulted in an approximately 1 log growth reduction independently of the antibiotic resistance pattern of the *S. aureus* strains used [33]. Histological evaluations of untreated and treated skin areas upon irradiation within 24 h did not show significant degree of necrosis or apoptosis [33]. 

Over 40 different virulence factors including a wide range of enzymes and toxins have been identified in *S. aureus*, which are involved in almost all processes from colonization of the host to nutrition and dissemination [34, 35]. However, in general, conventional antibiotics have no effect on inactivating these virulences. The activities of V8 protease, *α*-haemolysin, and sphingomyelinase expressed by epidemic MRSA16 were identified to be inhibited in a dose-dependent manner (1–20 *μ*M) by exposure to laser light in the presence of MB [36]. Moreover, inactivation of *α*-haemolysin and sphingomyelinase is not affected by the presence of human serum, indicating that PDT may be effective against these toxins in vivo [36]. The ability of PDT to reduce the virulence of MRSA, as well as effectively inactivating the organism, would represent a significant advantage over conventional antibiotic strategies.

Although there have been encouraging reports of the use of antimicrobial PDT to inactivate MRSA in large in vitro studies, there have been relatively few reports of their use to treat MRSA infection in vivo. And all the current in vivo studies are confined within local MRSA infection on rodent models. A mouse model of skin abrasion wound infected with bioluminescent strain of MRSA Xen31 was developed [37]. This bioluminescent strain allows the real-time monitoring of infection in mouse wounds. PDT was performed with the combination of a series of concentrations of photosensitizer polyethylenimine- (PEI-) ce6 and a series of doses of noncoherent red light 30 minutes after bacterial inoculation. PDT resulted in 2.7 logs of inactivation of MRSA as judged by loss of bioluminescence in mouse skin abrasion wounds and accelerated wound healing by 8.6 days compared with the untreated infected wounds [37]. A tetracationic Zn(II)phthalocyanine derivative was also shown to inactivate MRSA, inhibit regrowth, and accelerate wound healing by using the mouse skin abrasion model [38]. Simonetti et al. [39] established full-thickness wounds with diameter of 0.8 cm, which were then inoculated with 5 × 10^7^ CFU of MRSA in the back subcutaneous tissue of BALB/c and CD1 mice. A strong reduction of bacterial counts (3 logs) was observed in mice treated with RLP068/Cl and illumination in comparison with infected untreated mice 2 days after infection. By day 9, a comparable and significant reduction of bacterium and a complete reepithelialization were found in mice treated with RLP068/Cl or with antibiotic teicoplanin [39]. A 25-fold reduction in the number of epidemic MRSA16 treated with 100 *μ*g/mL of MB and 670 nm laser light (360 J/cm^2^) was achieved in another mouse skin wound model [40].

MRSA arthritis is another animal model chosen to test the effectiveness of PDT for MRSA infection in vivo. A murine MRSA arthritis model showed that approximately 30% of intra-articular leukocytes, mainly neutrophils, died immediately after PDT [41]. A further decrease in the number of intra-articular leukocytes and atrophy of the synovial tissue were seen 24 h after PDT. The isolated peripheral neutrophils presented significant affinity to Photofrin and showed significant morphological damage after PDT with Photofrin [41]. These results indicated that PDT might not be highly effective for treating MRSA arthritis, because intra-articular neutrophils and synovial tissue were also injured by PDT. In order to maximize bacterial inactivation and minimize inactivation of host neutrophils, an intra-articular injection of Photofrin instead of intravenous administration was used and the light dosimetry was optimized to treat arthritis induced by MRSA infection [42]. Each animal received a knee injection with MRSA (5 × 10^7^ CFU) followed 3 days later by 1 mg of Photofrin and 635 nm illumination with a range of fluences within 5 minutes. The greatest reduction of MRSA was seen with a fluence of 20 J/cm^2^, whereas lower antibacterial efficacy was observed with fluences that were either lower or higher. Consistent with these results, a significantly higher concentration of macrophage inflammatory protein-2 (a CXC chemokine) and greater accumulation of neutrophils were seen in the infected knee joint after PDT with a fluence of 20 J/cm^2^ compared to fluences of 5 or 70 J/cm^2^ [42]. These results indicate that PDT for murine MRSA arthritis requires appropriate light dosimetry to simultaneously maximize bacterial inactivation and neutrophil accumulation into the infected site, while too little light inactivates sufficient bacteria and too much light inactivates neutrophils and damages host tissue as well as bacteria and allows bacteria to grow unimpeded by host defense.

## 4. Modification on Charge and Structure of Photosensitizers

A potential photosensitizer for antimicrobial PDT must have appropriate photophysical properties, such as a large and long wavelength absorption band and a high quantum yield for the generation of both long-lived triplet excited state and cytotoxic ROS species. It also has to be water-soluble and must have a high affinity to microbial cells and a low affinity to host cells. These characteristics are strongly related to the presence of cationic charges in the molecular structure.

Several groups [9, 43, 44] observed that photosensitizer charge and structure might be important factors in determining the success of antimicrobial PDT, especially when applied on negative surface charge of microorganisms like Gram-negative bacteria. Meso-substituted tetrahydroporphyrin tetratosylat (BL1065) was reported to acquire the ability to bind both Gram-positive (MRSA) and Gram-negative bacterial cell envelope more strongly than the dianionic chlorine BLC1013, resulting in better efficiency of photoinactivation [45].

Foley et al. [46] demonstrated that replacement of the oxygen atom in photosensitizer 5-(ethylamino)-9-diethylaminobenzo[a]phenoxazinium chloride (EtNBA) with sulfur and selenium afforded thiazinium (EtNBS) and selenazinium (EtNBSe) analogues that had similar water solubility, lipophilic character, and uptaking rate. But this small change on the molecule gave EtNBS and EtNBSe better antimicrobial efficacy than their chalcogen analogue EtNBA mainly due to higher triplet quantum yield. Replacing the central oxygen atom with a somewhat heavier sulfur atom resulted in a small but significant increase in the triplet yield (0.03) and that as expected the replacement by a much heavier selenium atom resulted in a dramatic improvement in the triplet yield (0.78) [46]. It is well known that incorporating a heavier atom into a molecule with a low intrinsic intersystem crossing rate constant will increase the probability of such transitions roughly in proportion to the square of the spin-orbit coupling constant of the atom where the transition occurs [47].

In another report [48], two EtNBS derivatives were synthesized, each functionalized with a different side-chain end-group, alcohol or carboxylic acid. There were no significant changes in absolute quantum yield of singlet oxygen formation, and both derivatives were phototoxic to *S. aureus* 29213, but the carboxylic acid derivative was nontoxic to *E. coli* 25922. This suggests that small functional groups of photosensitizer could achieve Gram-type-specific phototoxicity through altering the photodynamic activity of photosensitizer and deserve further exploration in a larger number of representative strains of each Gram type including MRSA.

## 5. New Drug Delivery Strategies Design

For antimicrobial PDT to be of clinical use, effective delivery methods for both light and photosensitizers to the site of action are necessary. Due to limited light penetration through tissue, clinical antimicrobial PDT will be necessarily limited to areas of the body where light can be delivered relatively easily, such as the skin and body cavities, as opposed to systemic infections such as bacteremia [49]. In contrast to conventional high irradiance treatments, recent preclinical and clinical photodynamic studies have focused on low irradiance schemes [50–52], which consume less oxygen than high irradiance. Compared with light and oxygen delivery, photosensitizer delivery system seems much more complicated. Researchers focused on drug delivery strategies for efficient but specific therapy. 

Currently, photosensitizers under investigation at either a preclinical or clinical level are systemically administered after incorporation into lipophilic delivery systems, such as liposomes, oil emulsions, or cyclodextrin inclusion complexes in order to minimize precipitation in the bloodstream or aggregation in a polar milieu, which decreases PDT therapeutic efficiency [53, 54]. As for MRSA, an enhanced inactivation of MRSA by a liposome-delivered photosensitizer was demonstrated compared with the free dye [55]. Hematoporphyrin was embedded in fluid cationic vesicles composed of the monocationic lipid N-[1-(2.3-dioleoyloxy)propyl]-N,N,N-trimethylammonium methylsulfate, which yields an endocellular concentration of photosensitiser much higher, yet promotes a tighter binding and a more efficient photoinactivation of MRSA. 

The use of polymeric micelles as vehicles of photosensitizers is another very promising approach for photodynamic therapy [56–58]. The polymeric micelle delivery system may improve drug solubility and prevent the formation of aggregates in the aqueous medium. Compared to the use of liposomes, preparation of polymeric micelles can be much less expensive and simpler. In a recent study, photosensitizer hematoporphyrin was encapsulated with liposomes and micelles by the reversed-phase evaporation method, and both micelle and liposome delivered hematoporphyrin induced complete eradication of the Gram-positive pathogens including both MSSA and MRSA [59]. The hematoporphyrin dose completely eradicating pathogens using micelle and liposome was significantly lower than the dose required when using the nonencapsulated hematoporphyrin. The photodynamic inactivation effect of the hematoporphyrin encapsulated in polymeric micelles was superior to the hematoporphyrin encapsulated in liposomes at lower hematoporphyrin doses [59]. 

In a different approach, an optimised formulation (8.0% w/w poly(vinyl alcohol), 2.0% w/w borax) of hydrogel was synthesized with 1.0 mg/mL of the photosensitizers MB and meso-tetra(N-methyl-4-pyridyl)porphine tetra tosylate (TMP), both of which were found to be phototoxic to planktonic and biofilm-grown MRSA [60]. Furthermore, newborn calf serum, which was used to simulate the conditions prevalent in an exuding wound, did not adversely affect the properties of the hydrogels and had no significant effect on TMP-mediated photodynamic inactivation of MRSA, despite appreciably reducing the fluence rate of incident light. Topically applied to treat wound infection, hydrogels loaded with photosensitizers possess the ability to flow into and produce intimate contact with wounds even heavily exuding wounds, whilst their dilated structure allows for intact removal once the treatment is completed. These characteristics may facilitate clinical use of photodynamic therapy. 

## 6. Targeted Antimicrobial PDT of MRSA

One possible problem with the use of light activated antimicrobial agents is that the ROS produced during the process have the potential to damage neighboring host cells. There is, therefore, great interest in developing methods of targeting the photosensitizer of the infecting organism. The challenge in antimicrobial PDT is to find a therapeutic window, in which hazardous bacteria are efficiently inactivated without harming the surrounding tissue and disturbing the local microenvironment at a given concentration and light dose. The ability to confine activation of the photosensitizer by restricting illumination to the bacteria allows for a certain degree of selectivity towards these cells. Improved selectivity with preferential bacterial uptake of photosensitizer through modification of photosensitizer is another promising approach. To date, methods of targeting photosensitizers specifically to a certain type of microorganism include antibody conjugation [61, 62], attachment of peptides [63], employing bacteriophages [64], and taking advantage of the resistance mechanism of microorganisms [65].

Antibody conjugated with various photosensitizers was reported as a very promising targeting PDT [66–68]. As for antibacterial PDT, a lethal photosensitization of MRSA using an immunoglobulin G-tin(IV)chlorine e6 conjugate as the respective photosensitizer was reported [62]. A number of isotypes of immunoglobulin G bind through the Fc region to protein A, which is expressed and localized as a typical cell wall protein by quite few MRSA strains. The amount of protein A embedded in the cell wall areas can vary among these strains [69]. A close relationship between protein A amount and inactivation efficacy was observed in the use of the immunoglobulin G-tin(IV)chlorine e6 conjugate [62]. Despite many promising in vitro results, antibody targeting antibacterial therapy has only had little real success in either antibacterial PDT or cancer therapy. There are a number of problems associated with antibody-based photodynamic therapies, including difficulty to achieve specific antibodies that also display high affinity, inconsistent expression of target antigens, and difficulty to internalize antibodies by the same cells [70]. 

The possibility of using a bacteriophage to deliver the photosensitizer tin(IV)chlorine e6 (SnCe6) to a serial strain of *S. aureus* was also investigated [64]. Substantial inactivations of both MRSA and vancomycin-intermediate strains were achieved with low concentrations of the conjugate (1.5 *μ*g/mL SnCe6) and low light doses (21 J/cm^2^). Under these conditions, the viability of human epithelial cells in the absence of bacteria was largely unaffected. On the molar equivalent basis, the conjugate was more effective than the unconjugated SnCe6, and bacterial inactivation was not growth phase dependent. Furthermore, the conjugate was effective against vancomycin-intermediate strains even after growth in vancomycin [64]. These results indicated that a bacteriophage might be used to deliver a photosensitizer to a target organism, resulting in improving efficiency and specificity in inactivation of the MRSA and other organisms, which are desirable in the photodynamic therapy of infectious diseases. 

Another method was demonstrated to target MRSA by taking advantage of its most common resistance mechanism [65]. A specific enzyme-activated structure (*β*-LEAP) was developed, for which two phenothiazinium photosensitizers (EtNBS-COOH) were combined to the side chains of cephalosporin. The two photosensitizers were quenched in the uncleaved construct due to close proximity to each other, but were activated through cleavage of the lactam ring by beta-lactamase, which was synthesized only by resistant strains. The selectivity of *β*-LEAP was demonstrated through coculture experiments with human foreskin fibroblasts (HFF-1) and MRSA strain. There was only little nonspecific uptake of *β*-LEAP by the HFF-1 cells in the presence of MRSA, while the MRSA stain had far greater *β*-LEAP uptake [65]. This novel targeting strategy of the resistance mechanism itself has, besides the specificity for enzyme-mediated resistant microbia, the potential advantage to distinguish between human and microbial cells.

## 7. Microorganism Strain Selective and Antimicrobial PDT Resistant

Compared with traditional antibiotic therapy, microbes, including MRSA, rarely develop resistance to antimicrobial PDT. However, Grinholc et al. [71] recently demonstrated that biofilm not producing *S. aureus* strains was much more sensitive to PDT than to their slime-producing isolates. In addition, neither correlation between antibacterial PDT effectiveness and the antibiotic resistance pattern of the different strains, nor correlation between photodynamic inactivation efficacy and differences within proteins profiles could be demonstrated [71]. Possibly biofilm produced by bacterium that obstructs the photosensitizer penetrating into bacterial cells plays a role in resistance to PDT. The effect of extracellular slime on photodynamic inactivation of bacteria was also analyzed by another group [72], who reported that extracellular slime significantly influenced the sensitizer uptake by the *S. aureus* cells. However, biofilm nonproducing strains could also be found with elevated resistance to PDT, and strains with a similar uptake possess significantly different susceptibility to PDT [73]. The different uptake due to extracellular slime did not determine the strain dependence of PDT solely. 

Efflux mechanisms have been recognized as important components of microbial resistance of MRSA to various classes of antibiotics. NorA efflux pump as one of the multidrug resistance pumps (MDRs) has the ability to expel a variety of structurally diverse compounds [74]. The uptake levels of phenothiazinium-based sensitizers MB, TBO, and 1,9-dimethylmethylene blue (DMMB) by various strains of *S. aureus* were showed to be proportional to levels of NorA expression [75]. This suggested that MDRs were able to pump the photosensitizer out of the cells and thereby lessen the photoinactivity. However, the uptake level of non-phenothiazinium-based photosensitizer protoporphyrin diarginate was observed not to be affected by NorA expression levels [73]. And the MDR inhibitor reserpine did not affect the bactericidal activity either [73]. Therefore, the efflux mechanism might not influence the uptake level of all photosensitizers or the efficiency of MRSA inactivation. 

Despite numerous reports demonstrating that a variety of photosensitizers can be used to inactivate *S. aureus* strains including MRSA, some sensitizers show little or no bactericidal effect towards several strains [71]. The mechanism responsible for strain-dependent inactivation and photodynamic resistance has not yet been definitively identified.

## 8. Present Problems and Future Works

Due to the requirement that light should be delivered to the microorganism, indications for antimicrobial PDT for MRSA are the treatment of local, superficial skin and soft tissue infections and arthritis. Topically applied photosensitizer with subsequent irradiation has locally limited action of the PDT and side effects such as allergic contact sensitization and disturbance of the resident flora. Therefore, the severe side effects of systemic administration of conventional antibiotic for local MRSA infection are avoidable. 

In order to attain high antibacterial activity with topical antibacterial PDT, sufficient concentration of photosensitizer at site (within bacterial cells or attached to the cell membrane) is needed. A basic prerequisite for the effective use of antimicrobial PDT is the uptake and/or binding of the photosensitizer on the bacterial cell wall or plasma membrane. Thus the design of the molecular structure and the functional side chains of the photosensitizer [46, 48] and the charge [44], as well as the manner in which the photosensitizer is transported [20, 53–55, 59, 60], could influence the efficiency of antimicrobial therapy. Cationic photosensitizers with positive charge are usually more efficient than their neutral and negative charged analogues when they are used to inactivate Gram-positive and Gram-negative microorganisms. Significant alteration of the efficiency for inactivating Gram-positive and Gram-negative bacteria can be achieved through modifying benzo[a]phenothiazinium dyes with one atom and one side chain, respectively [46, 48]. Systemic administration of photosensitizers after incorporation into lipophilic delivery systems, such as liposomes, oil emulsions, or cyclodextrin inclusion complexes, can minimize precipitation in the bloodstream or aggregation in a polar circumstance, which reduces PDT therapeutic efficiency. Photosensitizers encapsulated in liposomes and micelles or loaded into hydrogel achieved better inactivation of MRSA for local application of PDT to inactivate MRSA in vitro. Additionally, combining cationic modification and delivery system of polymer was believed to increase the efficacy of inactivation [76].

Moreover, in order to improve specificity, targeting photosensitizers specifically to a certain type of microorganisms was tested. Those targeting systems which have shown promise in laboratory included chemical modification of the photosensitizer itself, drug delivery strategy optimization, the usage of antibodies and bacteriophage, and conjugation with traditional antibiotic [61–65]. As well as achieving better selectivity, another advantage of using a targeted photosensitizer is the increased antimicrobial efficiency. That is because, following binding of the targeted photosensitizer to the organism, subsequent irradiation results in the generation of ROS only in the vicinity of the pathogen and not at extraneous sites. Consequently, less photosensitizer needs to be applied, and because there is less attenuation of the incident light by unbound photosensitizer, a lower light dose can be used. However, a variety of disadvantages can hamper effective photodynamic inactivation. For instance, the very high molecular weight of such photosensitizer complexes may inhibit penetration of the upper layers of the epidermis needed for effective treatment of superficial skin MRSA infections. Also alterations of the binding epitopes on the protein surface of MRSA could result in a loss of antibody recognition and thus in a loss of photodynamic activity.

At present, it is still unknown whether resistance to PDT will be developed by MRSA. The number of photosensitizer molecules binding to the surface of MRSA cells is limited by biofilm formation and tunnel protein-deficient mutation, and active outward transport of photosensitizer can reduce photosensitizing efficiency toward MRSA [72, 74]. But in the studies from Grinholc et al. [71, 73], biofilm nonproducing strains could also be found among *S. aureus* strains with elevated resistance to PDT, and no association between photodynamic inactivation efficacy and the antibiotic resistance pattern (MDRs) of the different MRSA strains or the antibiotic-sensitive MSSA strains could be demonstrated. In addition to membrane structure and extracellular biofilm, cellular repair systems or concentration of antioxidant enzymes might also contribute to resistance. ROS inducing cellular necrosis and apoptosis play pivotal role in photodynamic bacterial inactivation. However, the production of ROS, particularly singlet oxygen, during irradiation occurs only precisely at the location of the photosensitizer. Singlet oxygen is only short lived in biological systems and in parallel possesses only a very limited diffusion distance (in pure water about 1 *μ*m, while no more than 50 nm in the vicinity of protein-rich lipid milieu) [77]. To date, it is uncertain whether MRSA is capable of developing resistance towards ROS through antioxidant enzymes activation or other possible mechanisms. Nevertheless, the mechanism responsible for resistance of certain MRSA strains and MSSA strains towards PDT thus needs to be definitively clarified in the future.

## 9. Conclusion

It can be said that the optimized physicochemical properties of photosensitizers as well as specific delivery systems will decide whether antimicrobial PDT for MRSA infection could be accepted as an alternative way to traditional antibiotic therapy. After further well-designed preclinical and clinical studies, this novel therapeutic approach for MRSA infection treatment may be established in clinical practices.
